# Impact of Variation in Amylose Content on Durum Wheat cv. Svevo Technological and Starch Properties

**DOI:** 10.3390/foods12224112

**Published:** 2023-11-13

**Authors:** Mike Sissons, Samuela Palombieri, Francesco Sestili, Domenico Lafiandra

**Affiliations:** 1 NSW Department of Primary Industries, Tamworth Agricultural Institute, 4 Marsden Park Road, Tamworth, NSW 2340, Australia; 2 Department of Agriculture and Forest Sciences (DAFNE), University of Tuscia, Via San Camillo de Lellis snc, 01100 Viterbo, Italy; palombieri@unitus.it (S.P.); francescosestili@unitus.it (F.S.); lafiandr@unitus.it (D.L.)

**Keywords:** durum, starch digestion, pasta quality, semolina, dough properties

## Abstract

Reserve starch, the main component of durum wheat semolina, is constituted of two glucan homopolymers (amylose and amylopectin) that differ in their chemical structure. Amylose is mainly a linear structure formed of α-1,4-linked glucose units, with a lower polymerization degree, whereas amylopectin is a highly branched structure of α-1,4-chains linked by α-1,6-bonds. Variation of the amylose/amylopectin ratio has a profound effect on the starch properties which may impact the wheat technological and nutritional characteristics and their possible use in the food and non-food sector. In this work a set of genotypes, with a range of amylose from 14.9 to 57.8%, derived from the durum wheat cv. Svevo was characterised at biochemical and rheological level and used to produce pasta to better understand the role of amylose content in a common genetic background. A negative correlation was observed between amylose content and semolina swelling power, starch peak viscosity, and pasta stickiness. A worsening of the firmness was observed in the low amylose pasta compared to the control (cv. Svevo), whereas no difference was highlighted in the high amylose samples. The resistant starch was higher in the high amylose (HA) pasta compared to the control and low amylose (LA) pasta. Noteworthy, the extent of starch digestion was reduced in the HA pasta while the LA genotypes offered a higher starch digestion, suggesting other possible applications.

## 1. Introduction

Starch is the main carbohydrate in the endosperm of wheat grain, it is deposited in granules and makes up for 70–80% of flour weight. Starch composition includes two types of polyglucans, amylose and amylopectin, generally with a 1:3 ratio. Amylopectin is characterised by the presence of glucose residues connected through α-(1,4)-bonds and with considerably shorter chains and several α-(1,6)-branches, whereas the amylose is mainly linear and consists of glucose residues connected through α-(1,4)-linkages to form long chains with a low number α-(1,6)-branches [1].

Amylose and amylopectin synthesis involve different classes of enzymes, with, a granule-bound starch synthase (GBSSI) involved in amylose synthesis, whereas amylopectin is produced by the coordinated action of different starch synthases (SSI, SSII, SSIII), starch-branching enzymes (SBEI, SBEIIa, and SBEIIb) and starch-debranching enzymes of isoamylase and limit dextrinase-type (ISA and LD) (reviewed in [2]).

The amylose/amylopectin ratio results in being a major contributor of the starch structure and its functional properties. The identification of genes involved in starch biosynthesis has enabled the possibility to manipulate the amylose/amylopectin ratio, through classical breeding, transgenesis, and mutagenesis [3,4,5,6,7]. 

Bread wheat possesses three GBSSI isoenzymes or waxy proteins, as they are commonly called, encoding genes that are present on the short arms of chromosomes 7A and 7D and on the long arm of chromosome 4A [8,9]. In durum wheat, as a consequence of the lack of the D genome, only two waxy proteins, Wx-A1 and Wx-B1 are present. Inactivation of one or two of these (one in durum wheat) homeologous genes results in the production of what are termed partial waxy wheats, whereas the term full waxy is associated with the silencing of all the three genes (two in durum) [10].

The starch from waxy wheat can be used as a source of blending in the preparation of products where high water absorption, greater viscosities, high digestibility, and low retrogradation rate are desired. In particular, the use of waxy wheat flour or semolina allows modulation of the rate and the extent of starch retrogradation, with the result of increasing the shelf-life of baked products, retarding bread staling, improving the stability of frozen foods, and the palatability of baked and sheeted-wheat products [11,12,13,14,15]. In addition, waxy starches, are hydrolysed rapidly and more completely than normal and high-amylose starches; thus, these can be used for the development of starch-based sweeteners and industrial alcohol, as in these products, quantitative conversion of starch to sugar is desirable [16].

Starch digestibility plays an important role in human health [17]. When foods are ingested a variable proportion of the starch remains undigested in the small intestine and reaches the large intestine where it is fermented by the gut bacterial microflora. This starch, which has been termed resistant starch, has been defined as the starch and products of starch digestion that are not absorbed in the small intestine of healthy individuals. Resistant starches behave as dietary fibre with major important health benefits including the prevention of the onset of cardiovascular disease, some types of cancer, obesity, and type 2 diabetes [18]. Some breeding programmes focus on the increase of amylose in the kernel because the tight association between amylose in the seed and resistant starch in derived foods is well ascertained (reviewed in [2]).

The suppression of two genes has been found to be involved in increasing amylose content in cereals, the starch synthase IIa (SSIIa) and the branching enzymes IIa (SBEIIa), while both have been investigated in bread and durum wheat. The starch synthase IIa (SSIIa) is involved in the synthesis of intermediate amylopectin chains with regard to the degree of polymerisation (DP) 12–24 by elongating short chains (DP ≤ 10) of amylopectin [19]. In durum and bread wheat the silencing of these genes has a strong effect on the composition and amount of starch in endosperms [20,21,22]. In detail, the phenotype is characterized by starch granules with a deformed shape, a high amylose content, and an increase in resistant starch inside the grain.

Introgression of the SSIIa null alleles in the durum wheat cultivar Svevo was obtained by Lafiandra et al. [23] with the result of a drastic increase of amylose content of roughly 89% compared to the control plant. 

The other important target to obtain high amylose wheat is the *SBEIIa* gene. Sestili et al. [24], were able to suppress *SBEIIa* genes and increase the amylose content in the durum wheat cultivar Svevo up to 75% through an RNAi approach. The TILLING strategy was very effective in the identification of point mutations following EMS treatment and has been used by different authors to generate knockout mutants in *SBEIIs* genes in durum wheat [4,25,26]. Slade et al. [4], reported an increased amount of amylose and resistant starch (47.4% and 6.21%, respectively) in the durum wheat mutants derived from the variety Kronos. Sestili et al. [26] obtained similar results in the cultivar Svevo (52.7% and 6.47%). Sissons et al. [27] used semolina obtained from the SSIIa and SBEIIa mutants to prepare spaghetti, with the objective of reducing the glycaemic index of pasta while maintaining acceptable technological properties. 

Given the number of studies described to modify the amylose content of durum wheat, there appear to be no studies comparing the impact on functional and starch digestion properties of a wide range in amylose content in a common genetic background, allowing for a better evaluation of the role of amylose content. Here dough properties, pasta quality, and in vitro starch digestion in pasta were evaluated in a set of starch durum wheat mutants with the same genetic background (cv. Svevo) but with different amounts of amylose (from 14.9 to 55%).

## 2. Materials and Methods

### 2.1. Plant Material and Field Trials

Durum wheat lines Svevo Wx (complete null and two partial genotypes), Svevo SSIIa, and Svevo SBEIIa were previously produced [22,23,26]. The five lines along with the control (durum wheat cv. Svevo) were grown in an open field at the Experimental Farm of the University of Tuscia, located in Viterbo, Italy (lat. 42°26′ N, long. 12°04′ E, altitude 310 m a.s.l.) in the 2017 season: Nitrogen fertilization (180 kg ha^−1^) was split into three applications: the first was given before sowing as di-ammonium phosphate (20% of total N applied), the second when the first node was detectable above ground as urea (50% of total N), and the third 25 days later as ammonium nitrate (30% of total N). Each of the six genotypes are abbreviated as follows: Svevo is the control; Svevo LA = Svevo low amylose; Svevo Wx4A = Svevo waxy null 4A; Svevo Wx7A = Svevo waxy null 7A; Svevo SSIIa = Svevo starch synthase mutant IIa; Svevo SBEIIa = Svevo starch branching enzyme mutant IIa. 

### 2.2. Semolina Analyses

Wheat was cleaned, conditioned to a water content of about 16.5% and left to moisten overnight. Standard milling was performed in a Buhler MLU 202 mill (Buhler, Utzwil, Switzerland) with three breaking and three sizing passages [28]. Semolina analyses were in duplicate and assessed for the following: swelling power (SP) [27]; protein determined by Dumas combustion using a Leco TruMax CN combustion nitrogen analyser (Leco Corp., St. Joseph, MI, USA) calibrated with sulfamethazine [29] expressed on a 14% moisture basis (mb); amylose content of the semolina and resistant starch (RS) measured using Megazyme kits (Deltagen Australia, Melbourne, Vic, Australia). Semolina colour was measured using a Minolta Chroma meter CR-410 detector (Biolab Australia, Sydney, NWS, Australia) calibrated with a white tile supplied by the manufacturer. Measurements were S-L* (brightness, 100 = white; 0 = black), S-a* (positive value is redness and negative value is greenness), and S-b* (positive value, yellowness; negative value, blueness). Flour water absorption, adjusted to 14% mb (FWA, 14% mb) was determined using a MicroDoughLAB (Perten Instruments, Sydney, NSW, Australia) fitted with a 4-g bowl, mixing at 120 rpm to target peak 650 FU in duplicate [27]. Dough mixing was also assessed using a Mixograph [30] with key parameters being mixograph development time (MPT) and resistance breakdown (RBD). The GlutoPeak test was performed on a GlutoPeak device (Brabender, Duisburg, Germany) [31] with three parameters, peak mixing time (PMT), torque, area (sum of zones 1–5). The main indices collected were the maximum torque (torque), corresponding to the peak occurring as gluten aggregates; the peak maximum time (PMT), corresponding to the time before torque falls off when the gluten begins to breakdown, while the area under the peak (or energy) was calculated by summation of parameters provided by the software (A(0–1) + A(1–2) + A(2–3) + A(3–4)) and expressed in arbitrary units (AU), all calculated by the software version 1.1.0). 

### 2.3. Pasta Analyses

Spaghetti was prepared as previously described [30] but with adjustment to water added to make the dough based on the water absorption of the semolina to account for the higher water absorption of high amylose flours [13]. For 1 kg of semolina, the amount of water added was for Svevo LA, 311 mL; Svevo Wx4A, 285 mL; Svevo Wx7A, 287 mL; Svevo, 290 mL; Svevo SSIIa, 333 mL; Svevo SBEIIa, 342 mL. Dried pasta was stored in sealed plastic bags at room temperature until required for analysis. All pasta samples were cooked to their fully cooked time (FCT), the time taken for the central starch core to disappear [28], and assessed for texture (firmness peak height and area); overcooking tolerance = 100 × (firmness at FCT – firmness at FCT plus 10 min overcooking/firmness at FCT); stickiness (peak height and area), cooking loss (CL%), and water absorption (WABS) as described previously [30]. For firmness and overcooking tolerance, 12 replicate tests were performed per sample, for stickiness, a minimum of four replicate analyses per sample, and for cooking loss and water absorption of pasta, duplicate analyses were collected. The colour of uncooked spaghetti strands was measured with a minimum of 4 replicate readings as DPL* (brightness), DPa* (redness), and DPb* (yellowness). Insufficient sample did not allow analysis of the cooked pasta. Supp

### 2.4. Starch Preparation and Analyses

Starch was isolated from semolina and used for analysis of viscosity using a Rapid Visco Analyser (RVA4) as described previously [32]. Starch digestion of the samples was determined as described previously [32]. An amount of 8 g spaghetti was cooked in 250 mL water to FCT, drained and cooled in water. Six spaghetti strands were trimmed to ~5 mm length. To standardize digestions an amount of spaghetti to give 90 mg of starch for each sample was subject to digestion. About 9–12 pieces were added to two 100 mL conical flasks (sample and one control—no enzymes added) to which 6 mL of pre-heated RO water was added and 5 mL of pepsin (Sigma P-6887 from gastric porcine mucosa; Sigma-Aldrich, Macquarie Park NSW, Australia) solution (1 mg/mL in 0.02 M HCl) except for control with 0.02 N HCL added. Flasks were incubated with shaking at 140 rpm for 30 min in a water bath held at 37 °C. To terminate the reaction, 5 mL of 0.2 M sodium acetate buffer (pH 6.0) was added to each flask followed by addition of 5 mL of pepsin (Sigma P-6887 from gastric porcine mucosa) solution and 5 mL of buffer to controls then incubated for 360 min at 37 °C. During the incubation, at intervals, a 0.1 mL aliquot was removed from the reaction mixture and mixed with 0.9 mL of ethanol (to terminate the enzyme reaction). This mixture was assayed for glucose using the Megazyme GOPOD reagent kit as per instructions (Deltagen, Kilsyth, Victoria, Australia). Absorbance at 510 nm was recorded using a UV mini-1240 Spectrophotometer (Shimadzu Scientific, Rydalmere, NSW, Australia). Glucose content (mg/mL) = corrected sample absorbance (test sample absorbance − control absorbance)/absorbance glucose standard.

The incremental area under the digestion curves (AUC) was calculated. Duplicate digestions were performed on separate days. 

Starch digestion data were fitted to a first-order equation:

*C*t = *C*∞ (1 − e^−*kt*^)

where *C*t is the percentage of starch digested at a given time (*t*), *C*∞ is the estimated percentage of starch digested at the end point of the reaction, and *k* is the starch digestion rate coefficient. In order to obtain the values of *k* and *C*∞, this equation can be transformed into a logarithm of slope analysis plot where there is a linear relationship between ln(d*C*t/d*t*) and *k* as follows:

ln(d*C*t/d*t*) = −*kt* + ln(*C*∞*k)*

*k* and *C*∞ are calculated from the slope (−*k*) and intercept (ln(*C*∞*k*)), respectively. 

### 2.5. Statistical Analysis

Data were analysed using the statistical programme GenStat version 17.1.0.14713 with a generalised linear model and the means were tested for significant differences by the least significant difference statistic (LSD), using *p* < 0.05. Data were checked for normality. 

## 3. Results and Discussion

### 3.1. Impact of Amylose Variation on Semolina and Dough Properties

The six Svevo-derived genotypes were developed to differ only in their amylose content, and this ranged from 14.9 to 58.9% (Table 1). Unfortunately, the Svevo LA while being the full waxy phenotype, with two genes mutated and consequent absence of both Wx4A and Wx7A proteins, had a higher amylose content than the expected (0–2%, typical for the waxy phenotype) [23] but due to seed impurity contamination from Svevo, the amylose content was 14.9%. The two partial waxy genotypes had similar amylose and a moderate decrease compared to Svevo. The two (HA) genotypes (Svevo SSIIa, Svevo SBEIIa) had significantly higher levels of amylose compared to Svevo as reported previously [27]. The glutenin subunit composition determined by SDS-PAGE revealed no differences in the high (*Glu-A1* null; *Glu-B1* 7 + 8) and low molecular weight glutenin subunits (data not shown) with differences only in their protein content arising during plant growth in the field (Table 1). 

Svevo LA swelling power was not elevated as would be expected in fully waxy wheat [33] as higher levels of amylopectin allow more starch granule swelling, indicating a loosely bonded micellar structure due to absence of amylose (Table 1). The two HA genotypes had lower swelling power, due to the minor amount of amylopectin with amylose acting as a diluent [34]. The RS content of the Svevo and partial waxy lines remained very low (<0.3%) until the amylose content exceeded ~44% then increased to 3.84% in the highest amylose genotype—an 18-fold increase over Svevo (Table 1). Increased RS in SSIIa and SBEIIa nulls was reported previously at similar levels in durum wheat [22,25]. The RS increase is a consequence of the high concentration of amylose in the starch inner structure that produces a highly organized and packed structure, physically resistant to amylolytic enzymes [35]. 

Semolina protein content varied amongst the genotypes grown in the field, which is typical of field grown wheat because there is a genotype by environmental interaction for grain protein content [36], although there was a trend towards higher protein in the HA genotypes. This is likely because these mutants had significantly lower starch content (Table 2) which would account for the increased percentage of protein also assumed by others, rather than more protein synthesis [22,25]. There were differences in the semolina colour between genotypes and compared to Svevo, with Svevo SBEIIa having a much higher yellowness but with lower S-a* values. These differences can be reflected in the different protein and ash contents as the two high amylose genotypes had more ash in the semolina [27] and this was also reported for a SBEIIa null [25]. Ignoring the FWA of Svevo LA, increasing the amylose content led to an increase in FWA, with a rapid increase as amylose exceeded 35%. This has been attributed to higher fibre content in high amylose flours due to the presence of more resistant starch [13] and other components like arabinoxylans and β-glucans [2], that were found to be increased in high amylose genotypes [5]. 

**Figure 1:**
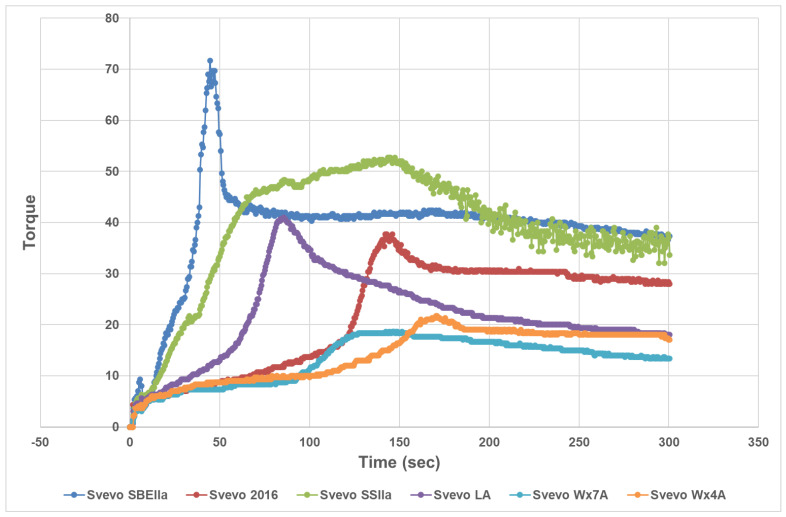
Glutopeak torque curves for Svevo samples.

Dough properties were assessed using two different instruments to provide an indicator of strength and stability. The mixograph showed that Svevo has moderately strong dough properties with MPT 3–5 min and a low RBD reflecting a stable dough after overmixing. The other genotypes were similar except for Svevo Wx4A with a very long MPT and the lowest RBD (Table 1). Svevo SBEIIa tended to show weaker dough properties compared to Svevo with the shortest MPT associated with weaker durum dough. The Glutopeak is a relatively new dough assessment tool [31] and the genotypes behaved quite differently with varying PMT, torque curves, and consequently areas under the curves (Table 1 and Figure 1). The weaker dough of Svevo SBEIIa was confirmed by the very short PMT of 47 s while Svevo Wx4A also showed a long PMT of 170 s aligned with a long MPT. The shape of the Glutopeak torque curve for Svevo SSIIa produced a broad curve which resulted in an inflated area. Hazard et al. [25] reported a limited effect of their SBEIIa mutation on gluten strength measured by gluten index and alveograph but the dough requires more water for hydration of the semolina as noted in this study. 

### 3.2. Impact of Amylose Variation on Pasta Technological Properties and Starch Pasting Viscosity

Important pasta quality traits were assessed using instrumental methods. Only the Svevo LA pasta had significantly shorter cooking time (FCT) than the other samples (except Svevo SSIIa) that were all similar and took longer for the central starch core to disappear (Table 2). A possible explanation for this is the high amylopectin content that contributes to a higher swelling power with shorter cooking time due to its ability to absorb water. Despite the HA pastas having the lowest total starch contents, which might be expected to mean having less starch to gelatinise, their FCTs were not significantly different to Svevo. The uncooked pasta colours (DPL*, DPa*, DPb*) did not reflect the trends shown in the semolina although Svevo SBEIIa was duller in appearance (lowest DPL*) compared to the other samples, probably due to a higher ash content [25,27]. For the cooked pasta firmness peak height, Svevo was the firmest with no differences between the other samples except the two partial waxy samples were significantly softer than all other samples. This trend was found in the firmness area except for the Svevo LA which was significantly lower than all other samples and this reflects the work in cutting through the strands. A lower firmness in complete waxy durum was reported previously [37] so this is not surprising but shows that measuring only the peak height can be misleading. Despite the higher protein content of the two HA genotypes, this did not translate into higher pasta firmness than Svevo although higher than the partial waxy sample, given the strong relationship between pasta firmness and protein content [38]. In contrast, Hazard et al. [25] reported an increased pasta firmness in their SBEIIa mutation due to higher protein and the ability of high-amylose starch granules to resist rupture on swelling. For the SSIIa mutation, Hogg et al. [39] noted a higher pasta firmness compared to the wild type. Previous work using reconstitution of durum components, where the starch was substituted with maize starch having varying amylose content, showed a tendency for firmness to increase with amylose content but was not strong [40]. 

The overcooking tolerance or resistance to firmness reduction is a good measure of cooking tolerance and pasta should resist being overcooked while still retaining al dente (having some firmness to the bite). The lower this value, the more tolerant the pasta is to firmness loss due to overcooking, which is desirable. It is clear that the Svevo LA had the worst overcooking tolerance losing a lot of its firmness after 10 min overcooking, consistent with a softer pasta typical of waxy wheat. For the Svevo LA we also find a significantly higher pasta stickiness peak height consistent with other reports [37] due to ease of solids lost from the pasta structure. The cooking tolerance of the other genotypes was similar to Svevo. There was a tendency for the high amylose pastas to have reduced stickiness peak height, but they were not significantly different to Svevo although less sticky than the partial waxy sample. However, for peak area both high amylose pastas were less sticky than Svevo (Table 2). A negative association between amylose content and stickiness was reported in reconstitution studies [27]. Compared to Svevo, both HA pastas had significantly higher cooking loss although not considered to be too high with acceptable levels (7–8%). A higher cooking loss was observed in SBEIIa nulls by Hazard et al. [25], but again they report around 6.4–6.7% results, similar to our own. The higher cooking loss could be related to the higher amylose content and its ability to leach out of the pasta structure during cooking. Higher amylose in the pasta also affected water uptake (WABS%), being significantly lower for the HA genotypes compared to Svevo and the partial waxy genotypes. This could be related to the reduced tendency for higher amylose starch granules to swell as they contain less amylopectin and have tightly packed granules that are more resistant to swelling. Hogg et al. [39] also noted their high-amylose SSIIa mutant made pasta that absorbed 16% less water, with a shorter cooking time with higher cooking loss. 

**Figure 2:**
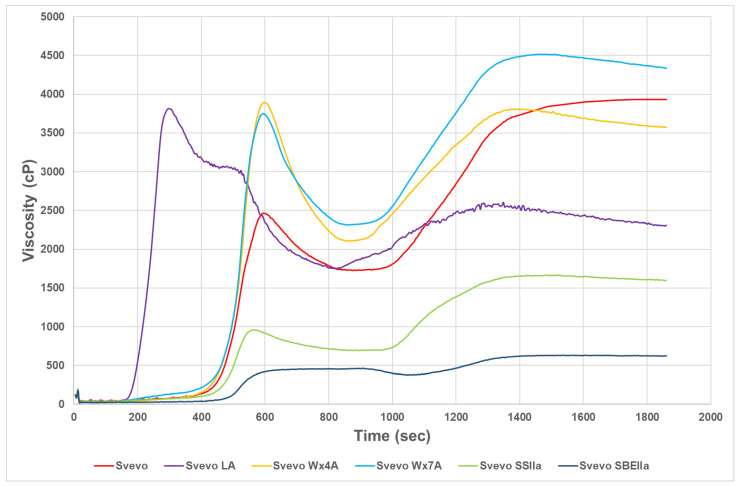
Starch pasting viscosities of Svevo samples.

The starch pasting curves for the Svevo samples are shown in Figure 2. During heating in the RVA, starch typically absorbs water causing the granules to swell and rupture followed by dissolution of the starch resulting in the slurry having an increased viscosity. A typical profile for Svevo LA like waxy durum was obtained with earlier peak time and lower final viscosity compared to Svevo, as reported previously [33]. The earlier viscosity is likely due to the more rapid swelling caused by more amylopectin compared to the other starches and by the reduction of the interactions between lipids and amylose chains. The lower final viscosity reflects the reduced retrogradation due to the lower amylose content being unable to maintain viscosity stability. The partial waxy genotypes showed similar profiles with higher peak viscosities but similar final viscosities to Svevo. The HA genotype RVA curves reflect the very low peak and final viscosities in high amylose starch similar to the findings by others for wheat [22,24,25]. This could be due the presence of ungelatinized starch in these higher RS genotypes or the reduced swelling in HA starches (low SP was observed) which would suppress the swelling of the starch granules during the heating program used for the RVA. However, the HA genotypes showed limited breakdown in viscosity that may offer benefits in some processes, also noted by Hazard et al. [25]. 

### 3.3. Impact of Amylose Variation on Pasta In Vitro Starch Digestion

**Figure 3:**
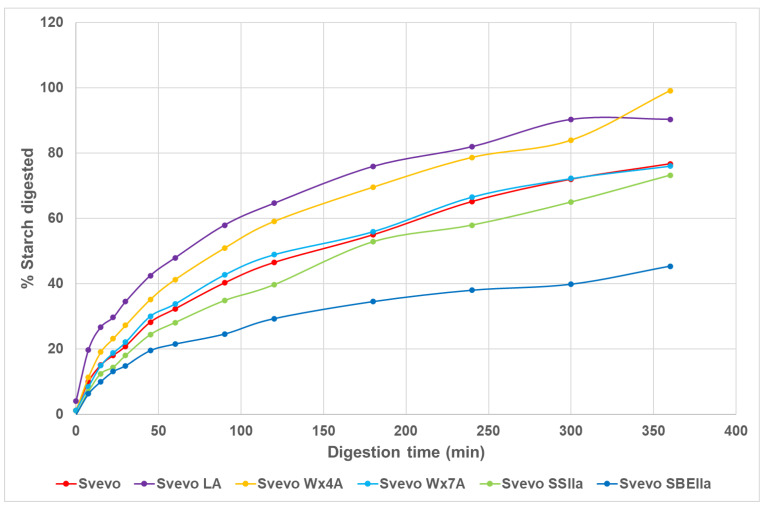
Digestibility curves of cooked pasta Svevo samples.

Typical starch digestion curves over the 360 min digestion are shown in Figure 3. Initially, digestion is rapid with Svevo LA showing faster digestion than the other samples and to a greater extent followed by Svevo Wx4A. After about 50 min into the digestion, the rate slows with the data following an exponential curve, so there are two different digestion rates typical for starch digestion in pasta. This is caused by the compact microstructure of pasta where the starch granules are embedded in a gluten matrix [41]. After 50 min a clear separation of some of the digestions curves occurs; for example, both Svevo LA and Svevo Wx4A curves are above the others reflecting a greater extent of digestion (area under the curve), while the two HA pasta curves show a much lesser extent of digestion (Figure 3 and Table 3). Svevo and Svevo Wx7A have similar curves while Svevo SSIIa is below these curves and SBEIIa has the lowest curve. The kinetics of these digestions can be quantified for the two phases of the digestion curve as rate constants k_1_, k_2_ and extent of digestion, C∞ % I and C∞ % II (Table 3). For all samples except SBEIIa, the C∞ % II values were between 80–100% with the SBEIIa having a much lower predicted digestion of 45.2%. These data align with the area under the digestion curves, with the higher amylose genotypes having less of their starch digested (lower area under the curve). Relative areas to Svevo show that Svevo LA and Svevo Wx4A are digested to a greater extent and Svevo SSIIa and SBEIIa the least, with the later much lower compared to Svevo. The initial rate k_1_, is faster than the second rate k_2,_ with Svevo LA having a faster k_1_ and k_2_ but with subtle differences between genotypes for k_2_. Interestingly, the Svevo SBEIIa has a higher k_1_ and k_2_ than all the other samples except Svevo LA. It was shown that to lower the in vivo glycaemic index in pasta only the Svevo SBEIIa was able to do this [27], indicating that relatively low in vitro starch digestion is needed.

## 4. Conclusions 

New genotypes with a Svevo durum wheat background were developed with the same glutenin composition but with variation in their starch amylose content from 14.9 to 57.8%. This permitted a good assessment of the impact of amylose variation on semolina dough, pasta properties, and on the in vitro starch digestion of the pasta. The general findings show that going from the lowest to highest amylose content, decreased the semolina swelling power (~52%), starch peak viscosity (~89%), and pasta stickiness area (~52%), while increasing the semolina water absorption (with the exception of Svevo LA) (~14.5%) and the pasta resistant starch (~66 fold). There was an indication that the dough properties of Svevo SBEIIa had weaker dough than Svevo but the HA pasta had acceptable pasta quality, with sensory analysis still needed to determine consumer preference for this kind of HA pasta with superior nutritional value. Importantly, the extent of starch digestion was reduced in the HA pasta, especially the Svevo SBEIIa, while Svevo LA offers a higher starch digestion more suited to other product applications. 

## Figures and Tables

**Table 1:** Semolina and dough properties of Svevo genotypes.

Genotype	Sowing Year	Amylose (% dmb)	SP		Protein * (14% mb)	S-L*	S-a*	S-b*	FWA% (14% mb)	MPT (Min)	RBD	PMT (s)	Torque (AU)	Area
Svevo LA	2016	14.9 ^a^	10.6 ^d^		12.8	83.4 ^b^	−1.4 ^d^	20.3 ^b^	67.6 ^d^	4.8 ^b^	58 ^c^	85 ^b^	41.7 ^d^	1833 ^a^
Svevo Wx4A	2016	30.0 ^b^	10.9 ^d^		10.9	84.2 ^d^	−1.8 ^c^	21.3 ^c^	58.7 ^a^	7.6 ^c^	13 ^a^	170 ^d^	21.7 ^b^	2132 ^b^
Svevo Wx7A	2016	31.7 ^c^	11.5 ^d^		11.0	83.4 ^b^	−0.8 ^e^	17.9 ^a^	59.4 ^b^	4.6 ^b^	75 ^c^	143 ^c^	18.7 ^a^	1716 ^a^
Svevo	2016	34.0 ^d^	8.9 ^c^		13.4	84.9 ^e^	−2.5 ^b^	24.0 ^e^	62.0 ^c^	4.8 ^b^	34 ^b^	145 ^c^	37.7 ^c^	2424 ^c^
Svevo SSIIa	2016	43.5 ^e^	6.6 ^b^		14.7	81.7 ^a^	−1.7 ^c^	22.6 ^d^	74.2 ^e^	3.9 ^ab^	68 ^c^	144 ^c^	53.0 ^e^	6016 ^d^
Svevo SBEIIa	2017	57.8 ^f^	5.1 ^a^		15.9	82.2 ^a^	−3.4 ^a^	31.0 ^f^	77.4 ^f^	2.5 ^a^	72 ^c^	47 ^a^	72.3 ^f^	1953 ^ab^
*p* < 0.005		<0.001	<0.001			<0.001	<0.001	<0.001	<0.001	<0.001	<0.001	<0.001	<0.001	<0.001
LSD		1.33	1.41			0.53	0.15	0.34	0.44	1.37	20.35	7.89	2.37	292

SP = swelling power; S-L* = semolina brightness; S-a* = semolina redness-greenness; S-b* = semolina yellowness; FWA = farinograph water absorption; MPT = mixograph development time; RBD = resistance breakdown; PMT = Glutopeak peak mixing time; * Typical st dev 0.015. Alike superscript letters in the same column are not significantly different, *p* < 0.05.

**Table 2:** Pasta properties of Svevo genotypes.

Genotype (Amylose%)	Sow Year	RS (% dmb)	TS (% dmb)	DPL*	DPa*	DPb*	FCT (s)	Firmness PH (g)	Firmness Area (g/s)	Overcooking Tolerance	S-PH (g)	S-Area (g/s)	CL%	WABS%
Svevo LA (14.9)	2016	0.11	70.2	65.6	3.5	32.9	551	1104	356	74	27.6	11.8	5.8	142
Svevo Wx4A (30)	2016	0.22	73.9	67.5	3.0	34.6	680	826	401	51	23.0	12.8	5.5	156
Svevo Wx7A (31.7)	2016	0.20	74.5	66.9	3.1	35.2	675	864	439	50	19.9	10.6	5.4	154
Svevo (34)	2016	0.73	73.4	70.1	0.3	44.6	664	1334	615	52	17.0	9.3	4.6	146
Svevo SSIIa (43.5)	2016	2.06	67.3	67.0	2.2	38.3	578	1125	566	51	14.7	5.4	6.9	123
Svevo SBEIIa (57.8)	2017	7.36	65.3	64.5	1.5	39.9	675	1124	544	49	15.9	5.7	6.6	120
*p* < 0.005		<0.001	<0.001	<0.001	<0.001	<0.001	0.010	<0.001	<0.001	<0.001	<0.001	<0.001	<0.001	<0.001
LSD		0.19	1.8	1.30	0.58	1.34	102.6	39.19	21.48	2.0	2.29	1.90	0.52	3.58

RS% = resistant starch; TS = total starch; DP-L* = dry pasta lightness; DP-a* = dry pasta redness; DP-b* = dry pasta yellowness; FCT = fully cooked time; S-PH = stickiness peak height; CL% = cooking loss; WABS% = water absorption.

**Table 3:** Kinetic parameters of digestibility for the Svevo pasta genotypes.

		Kinetics					
Genotype	Amylose%	C∞ % I	k_1_	C∞ % II	k_2_	Total Area under Digestion Curve	Normalised Area
Svevo LA	14.9	50.4	0.0402	92.8	0.0097	25,621	1.29
Svevo Wx4A	30.0	49.6	0.0278	101.3	0.0069	23,575	1.19
Svevo Wx7A	31.7	44.1	0.0239	79.9	0.0076	19,590	0.99
Svevo	34.0	38.9	0.0276	83.2	0.0065	19,851	1.00
Svevo SSIIa	43.5	35.4	0.0255	81.0	0.0057	18,652	0.94
Svevo SBEIIa	57.8	24.3	0.0347	45.2	0.0083	12,231	0.62

## Data Availability

Data is available upon request.
